# Pregnant women carrying microcephaly foetuses and Zika virus contain potentially pathogenic microbes and parasites in their amniotic fluid

**DOI:** 10.1186/s12920-016-0242-1

**Published:** 2017-01-11

**Authors:** Diogo Antonio Tschoeke, Louisi Souza de Oliveira, Luciana Leomil, Amilcar Tanuri, Fabiano Lopes Thompson

**Affiliations:** 1 Instituto de Biologia, CCS, Laboratório de Microbiologia, Anexo ao Bloco A, Universidade Federal do Rio de Janeiro, Ilha do Fundão, Rio de Janeiro, 21941-902 Brazil; 2 Laboratório de Sistemas Avançados de Gestão de Produção-SAGE-COPPE, Centro de Gestão Tecnológica-CT2, UFRJ, Rio de Janeiro, RJ Brazil; 3 Instituto de Biologia, CCS, Laboratório de Virologia Molecular, Bloco A, Ilha do Fundão, Universidade Federal do Rio de Janeiro, Rio de Janeiro, RJ Brazil; 4 Núcleo em Ecologia e Desenvolvimento Sócio-Ambiental de Macaé (NUPEM), Universidade Federal do Rio de Janeiro, Macaé, RJ Brazil

**Keywords:** Zika Virus, Metatranscriptomics, Microbiome, Microchepaly

## Abstract

**Background:**

Microcephaly has become a major public health problem in Brazil. The total number of newborns with microcephaly was reported to be >4000 in June 2016. Studies suggest that Zika Virus is a major cause of new microcephaly cases in Brazil. Inside the uterus, the foetus is surrounded by the Amniotic Fluid, a proximal fluid that contains foetal and maternal cells as well as microorganisms and where Zika Virus was already found.

**Case presentation:**

A previous study reported the presence of the Zika Virus in the amniotic fluid (collected in the 28th gestational week) of two pregnant women carrying microcephaly foetuses in Brazil. The virus was detected by means of real-time PCR and metatranscriptomic analysis. We compared the microbiome of these two cases with metatranscriptomic sequences from 16 pregnant women collected at various times in their pregnancies

**Conclusion:**

Several strains of bacteria (e.g., *Streptococcus* and *Propionibacterium*) found in Amniotic Fluid may be involved in neurological diseases. When the foetus is infected by the Zika Virus, due to neurological damage, they do not move inside the uterus, thus changing the Amniotic Fluid environment, potentially leading to secondary problems. Zika infection could also lead to an immunodeficient state, making bacterial colonization of the foetuses easier. An altered microbial composition during pregnancy may also result in harmful secondary metabolite production from certain microbes that further impair foetal brain development. However, these observations of potentially harmful microbial species are correlations and thus cannot be assumed to be causative agents of (microcephaly) disease. In our study, microbial and parasitic diversity of the Amniotic Fluid was lower in patients infected by ZIKV, compared to that of Prenatal and Preterm controls. The present study was a first attempt to shed light on the microbial and parasitic diversity associated with ZIKV-infected pregnant women bearing microcephaly foetuses, and the presence of diverse microbial and parasite communities in the Amniotic Fluid suggests a poor health status of both the pregnant women and the foetuses they carry.

**Electronic supplementary material:**

The online version of this article (doi:10.1186/s12920-016-0242-1) contains supplementary material, which is available to authorized users.

## Background

Microcephaly has become a major public health problem in Brazil. The total number of newborns with microcephaly was reported to be >4000 in June 2016. Several etiologic agents have been associated with microcephaly including genetic disorders (e.g., autosomal recessive microcephaly, Aicardi–Goutières syndrome, chromosomal trisomy, Rett syndrome, and X-chromosomal microcephaly), maternal malnutrition, drug and chemical intoxication (e.g., alcohol, cocaine, antiepileptic drugs, lead/mercury intoxication and radiation) and transplacental infections by viruses or bacteria [1]. Recently, a study showed that Zika Virus (ZIKV) induces cell death in human neural stem cells and thus impairs the formation of neurospheres in culture. This finding suggests that ZIKV is a major cause of new microcephaly cases in Brazil [2]. Inside the uterus, the foetus is surrounded by the Amniotic Fluid (AF), a proximal fluid that contains foetal and maternal cells as well as microorganisms [3–8]. The most common bacterial species found in the AF of women who undergo preterm labour with intact membranes are *Ureaplasma urealyticum*, *Fusobacterium* sp., and *Mycoplasma hominis* [5]. DiGiulio [6] also reported that *Sneathia*, *Bacteroides*, *Prevotella*, *Streptococcus*, *Leptotrichia*, *Peptostreptococcus*, *Escherichia*, *Gardnerella*, *Bacillus*, *Bergeyella*, *Citrobacter*, *Delftia*, *Lactobacillus*, *Neisseria*, *Clostridiales* and *Staphylococcus* were identified in the AF of women in preterm labour.

## Case presentation

A recent study has reported the presence of the Zika Virus in the amniotic fluid (collected in the 28th gestational week) of two pregnant women carrying microcephaly foetuses in Brazil [9]. The virus was detected by means of real-time PCR and metatranscriptomic analysis. The aim of the present study was to analyse the microbial (prokaryotic and eukaryotic) and parasitic diversity in the AF of these two cases (patients 1 and 2). Additionally, we compared these two cases with metatranscriptomic sequences from 16 pregnant women collected at various times in their pregnancies (four Prenatal samples [18–24 weeks], six late Preterm samples [34–36] weeks, and six Term samples [39–40 weeks]) [8].

The Illumina sequences obtained from these two cases [9] and the 16 metatranscriptomes used as controls [8] were pre-processed using Prinseq software to remove reads smaller than 35 bp and sequences with quality scores lower than Phred 20. The PEAR program (Paired-End read mergeR) [10] was used to merge and extend the paired-end Illumina reads using the default parameters, with max-overlap = 400 bp. To remove Human sequences, the extended reads were analysed using the Deconseq program [11], against Human Genome (Assembly version 37), with relaxed parameters (Identity and Coverage = 70%). The Non-human reads resulting from the previous step were analysed against the non-redundant GenBank Nucleotide Database (nt-db) (34,295,694 sequences), using BLASTn software (limited to the five best hits and e-value = 1e-5). The BLASTn results were processed using MEGAN 6 software, and the taxonomic assignment of the sequences was obtained using the Last Common Ancestor method with default parameters [12]. Sequences generated by Kamath-Rayne and colleagues were used as the three controls (I.E., Prenatal, Preterm and Term) [8]. Control sequences were processed following the same protocol used for the previous sequences (Patients 1 and 2). The Shannon and Simpson indices were calculated using the Vegan package from R language, using genus counts. The statistical significance of the results was evaluated using multiple T tests with a significance level of alpha = 0.05. To test the hypothesis that the taxonomic composition of the AF was the same in the four sample types (two infected Zika patients, Prenatal, Preterm and Term), Permutational Multivariate Analysis of Variance (PERMANOVA) was performed using the “adonis” function of the Vegan package [13] (Bray-Curtis distances and 999 permutations). A nonmetric multidimensional scaling (NMDS) analysis of the tabulated data (genus abundance) was performed using the metaMDS function in the Vegan R package to determine if the samples would group together.

**Fig. 1:**
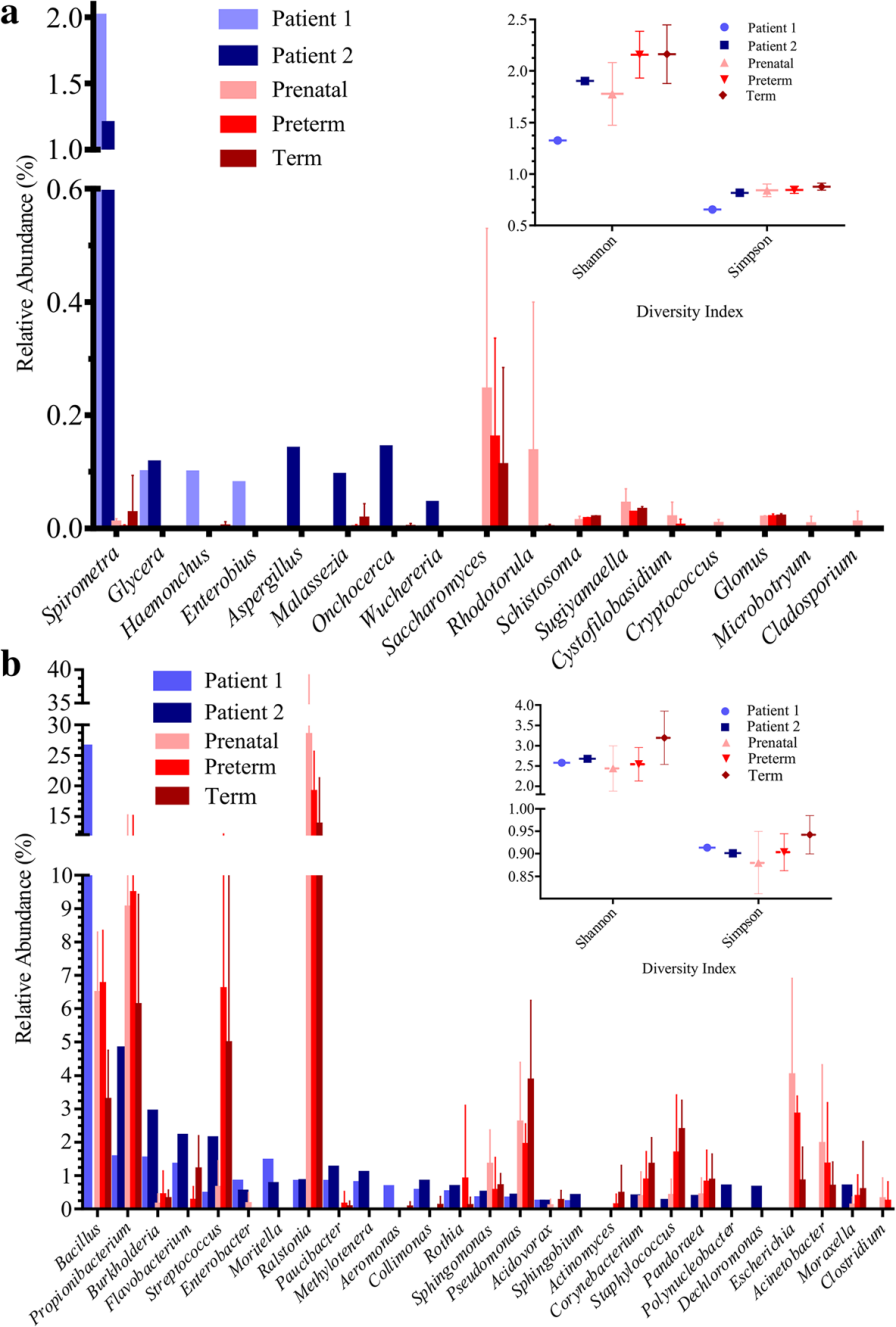
Relative abudance of the major taxonomic groups identified by MEGAN. **a** Eukaryotic groups (microbes and parasites) and **b** Bacteria in patients 1 (*light blue*); 2 (*dark blue*) and Controls (*red collors*). The Shannon and Simpson index calculated for Bacteria and Eukariotic domains, using Vegan R package, are shown in a right upper box. Controls, mean (±Standard Deviation) obtained from AF samples of 16 pregnant women collected in different times (“Prenatal”, “Late Preterm” and “Term”) deposited and available in SRA NCBI section (http://www.ncbi.nlm.nih.gov/bioproject/PRJNA281986). The Shannon and Simpson index were calculated with “Vegan” package from R language, using genus counts. In total, 170,928 and 166,717 eukaryotic sequences showed no hits or were not taxonomically assigned by MEGAN in samples from the patients 1 and 2, respectively. Unidentified Bacterial sequences were the most abundant group found in both samples (3483 reads in the sample from patient 1 and 6538 reads in the sample from patient 2). Shannon and Simpson indexes, for Eukariotic species (Fig. 1a), showed that Controls were more diverse than the two Zika infected AF samples, altough only the comparissons between Zika patients and Term Control (considering Simpson index); and Zika patients and Preterm Control (considering Shannon index) were statistically significant (Additional file 2: Table S2). The PERMANOVA analysis showed a statistical diference in the Eukaryotic composition between the samples types (Additional file 3: Table S3). Zika positive AF patients 1 and 2 were more diverse (Shannon index = 2.578 and 2.678; Simpson index = 0,91 and 0,90) than Prenatal and Preterm Controls samples (Shannon index = 2.44; Simpson index = 0.88 and Shannon index = 2.54; Simpson index = 0.9, respectivelly) (Fig. 1b). The Term samples were more diverse than Zika samples (Shannon index = 3.19; Simpson index = 0.94), however the difference were not statically significant in any comparisson (Additional file 2: Table S2). The PERMANOVA showed a statistical diference in the Bacterial species composition between the type of the samples (Additional file 3: Table S3), even as the NMDS analysis also showed that Zika patients clustering together, a part from the three controls groups (Additional file 4: Figure S1)

The Illumina sequencing of the AF from the two ZIKV patients resulted in 7,504,100 and 8,235,773 pair-end sequences. After pre-processing, sequence collapse, and removal of human sequences, the remaining 810,376 and 1,064,296 sequences were subjected to BLAST and MEGAN analyses. Fractions of these sequences were identified as Eukaryotes (157,075 and 253,852), Bacteria (19,074 and 24,826), and Viruses (611 and 2004), for patients 1 and 2, respectively. The most abundant eukaryotic group was the tapeworm *Spirometra* [3138 (1.99%) reads from patient 1 and 3018 (1.19%) reads from patient 2] (Fig. 1a; Additional file 1: Table S1); these sequences were related to the sequences annotated and deposited by Bennett and colleagues [14]. The tapeworm *Spirometra* is responsible for sparganosis and human brain lesions [15]. Spirometra is acquired by the ingestion of raw or undercooked meat from snakes or frogs, untreated drinking water, or raw flesh in traditional poultices. Sequences related to other fungi and parasites (e.g., *Onchocerca*, *Wuchereria*, and *Enterobius*) were also found. Except for *Onchocerca*, which can cause blindness, none of these parasites appear to have neural tropism [16].

*Bacillus* was the most common group in the AF of patient 1 [4993 (26.2%) reads] (Fig. 1b). The five most abundant groups found in both patients were *Propionibacterium* [286 (1.5%) and 1183 (4.8%) reads], *Burkholderia* [280 (1.47%) and 713 (2.87%) reads], *Flavobacterium* [244 (1.28%) and 533 (2.15%) reads], *Streptococcus* [78 (0.41%) and 515 (2.1%) reads], and *Paucibacter* [146 (0,77%) and 295 (1.19%) reads] (Fig. 1b). *Enterobacteria* phage phiX174 sequences were found in both patients [537 (87.9%) and 625 (31.19%) reads]. In contrast, *Ralstonia* was the most abundant genus in the Controls [= ~ 2700 (= ~ 20%) reads].

## Conclusions

Several strains of bacteria (e.g., *Streptococcus* and *Propionibacterium*) found in AF may be involved in neurological diseases. Amniotic fluid and the placenta contain different types of bacteria, particularly in pre-term birth cases [7, 17]. A rich placental microbiome has been observed in normal-term pregnancies. This microbiome likely makes important metabolic and immune contributions to the growing foetus [18]. Due to neurological damage, a foetus infected by the Zika Virus does not move inside the uterus, thus changing the AF environment, potentially leading to secondary problems. Zika infection could also lead to an immunodeficient state, making bacterial colonization of the foetuses easier. In prior studies, preterm infants with neonatal necrotizing enterocolitis showed lower microbial diversity compared to controls [19]. Similarly, disruption of the microbial community by diarrhoea from *Clostridium difficile* also resulted in a decrease in diversity in the faecal microbiome [20]. However, previous studies comparing the bacterial diversity in patients with immuno-deficient syndrome (HIV) showed that there is an increase in bacterial diversity in HIV-positive patients compared to HIV-negative patients [21, 22]. In our study, microbial and parasitic diversity of the AF was lower (*p* < 0.05) in patients infected by ZIKV, compared to that of Prenatal and Preterm controls (Additional file 2: Table S2﻿; Additional file 3: Table S3). *Propionibacterium* was found in women with preterm premature rupture of membranes during pregnancy [6]. This genus was the second most abundant in patients 1 and 2, suggesting an altered environment for foetal development. Moreover, the AF from the two ZIKV patients clustered apart from control samples in the present study (Additional file 4: Figure S1). An altered microbial composition during pregnancy may also result in harmful secondary metabolite production from certain microbes that further impair foetal brain development. However, these observations of potentially harmful microbial species are correlations and thus cannot be assumed to be causative agents of (microcephaly) disease. Differences in microbial diversity could be attributed to changes in the AF over the course of the pregnancy rather than to Zika infection. The present study was a first attempt to shed light on the microbial and parasitic diversity associated with ZIKV-infected pregnant women bearing microcephaly foetuses. The presence of diverse microbial and parasite communities in the AF suggests a poor health status of both the pregnant women and the foetuses they carry.

## Additional files

Additional file 1: Table S1. Similarity count table of sequences classified as Spirometra. (XLSX 14 kb)
Additional file 2: Table S2. Multiple T Teste performed comparing the diversity index (Shannon and Simpson) between AF Zika samples and Controls Groups. (XLSX 11 kb)
Additional file 3: Table S3. Adonis (PERMANOVA) results of taxonomic composition of AF metatranscriptomics (XLSX 11 kb)
Additional file 4: Figure S1. Nonmetric multidimensional scaling of bacterial genus frequence of Zika AF and Controls Samples AF. There was a distinction between the samples (Zika versus Controls), indicating that there is a difference in genera composition between the samples. (TIF 425 kb)

## Abbreviations

AF: Amniotic fluid
HIV: Imuno-deficient syndrome
ZIKV: Zika Virus
