# Supplementary figures and images for: Pregnant women carrying microcephaly foetuses and Zika virus contain potentially pathogenic microbes and parasites in their amniotic fluid
Source: BMC Med Genomics. 2017 Jan 11;10:5. doi: 10.1186/s12920-016-0242-1 (PMC5225515; doi:10.1186/s12920-016-0242-1)

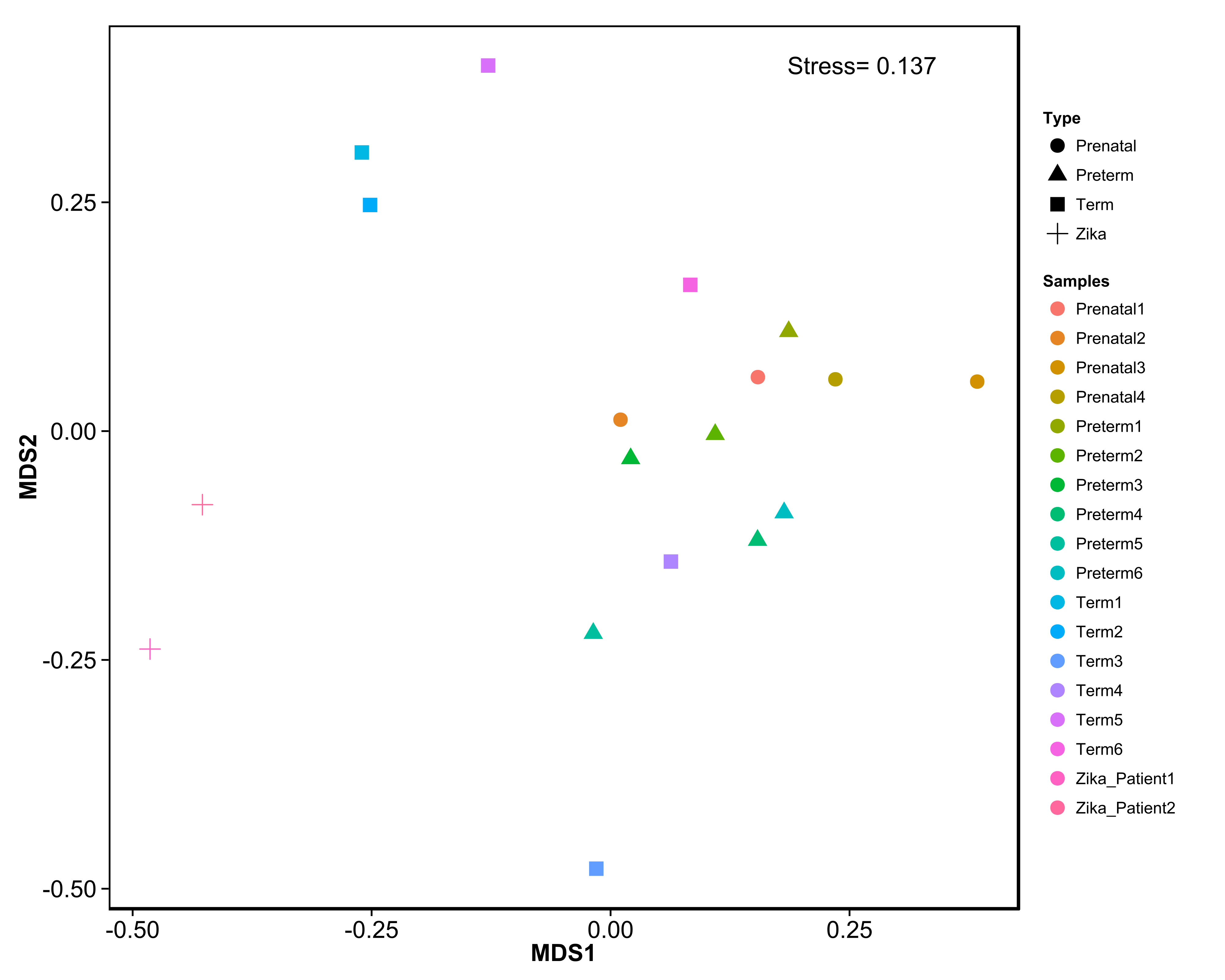

Supplement: Additional file 4: Figure S1. — Nonmetric multidimensional scaling of bacterial genus frequence of Zika AF and Controls Samples AF. There was a distinction between the samples (Zika versus Controls), indicating that there is a difference in genera composition between the samples. (TIF 425 kb) [file 12920_2016_242_MOESM4_ESM.tif]
